# Posterior lumbar fusion with and without interbody fusion in isthmic spondylolisthesis: a systematic review and meta-analysis

**DOI:** 10.1007/s10143-025-03703-x

**Published:** 2025-07-28

**Authors:** Kayoumars Azizpour, Sverre J. Broekman, Wilco C. Peul, Carmen L. A. Vleggeert-Lankamp

**Affiliations:** 1 Department of Neurosurgery, Leiden University Medical Center, Albinusdreef 2, 2300 RC, Leiden, 0031 71 5262109 The Netherlands; 2 Department of Neurosurgery, Haaglanden Medical Center, The Hague, The Netherlands; 3 Alrijne Hospital, Leiden & Leiderdorp, The Netherlands; 4 Department of Neurosurgery, Spaarne Hospital, Haarlem/Hoofddorp, The Netherlands

**Keywords:** Spine, Spondylolisthesis, Isthmic, Spondylolytic, Decompression, Laminectomy, Fusion, Spondylodesis

## Abstract

**Background:**

Isthmic spondylolisthesis is caused by a lesion in the pars articularis resulting in forward slippage of the vertebral segment relative to the caudal vertebral segment. Patients can develop neurogenic claudication or radiculopathy in case degeneration progresses. Nerve decompression is usually accompanied by spondylodesis: two frequently performed procedures are posterolateral fusion (PLF) and posterior lumbar interbody fusion (PLIF).

**Methods:**

Studies from four databases (PubMed, Embase, Web of Science, COCHRANE Library and Emcare) were included that compared clinical outcomes of posterolateral fusion (PLF) versus posterior lumbar interbody fusion in patients with isthmic spondylolisthesis. For Oswestry Disability Index (ODI) pooled point estimate and the 95% confidence interval (CIs) was derived using the random-effects model.

**Results:**

Fourteen studies met the inclusion criteria of which four were randomized controlled trials (RCT), six were retrospective, and four were prospective cohort studies (two with historical control). The pooled results for ODI yielded no difference between PLF and PLIF with an estimated pooled mean difference of -0.29 (95% CI [-0.83, 0.26], *P* = 0.45). A significant difference in favour of PLIF was demonstrated regarding SF-36 (1/3 studies) and VAS back pain (1/7 studies). None of the studies reporting Roland Disability Scores, Visual Analogue Scale (VAS) leg pain and (adjusted) Japanese Orthopedic Association score displayed a significant difference. Fusion rate was reported in favour of PLIF (2/6 studies).

**Conclusions:**

Our results suggest that both PLF and PLIF are adequate options for the treatment of isthmic spondylolisthesis. However, included studies gave insufficient data on possible confounding factors like age, degeneration status, grade of slippage, disc height, smoking, and facet joint condition. Therefore, there is not enough evidence to support either PLF or PLIF as the best treatment option. Large multicentre RCTs with proper adjustment for confounding factors and clearly defined inclusion and exclusion criteria should determine whether the two treatment modalities are indeed comparable.

**Registration:**

PROSPERO 2022 CRD42022338983.

**Clinical trial number:**

Not applicable.

**Supplementary Information:**

The online version contains supplementary material available at 10.1007/s10143-025-03703-x.

## Introduction

Lumbar spondylolisthesis is caused by forward slippage of a vertebral segment onto the vertebra below it. One of the causes of lumbar spondylolisthesis is a fracture in the pars articularis, resulting in isthmic spondylolisthesis [1]. In a cross-sectional study, Kalichman et al. reported a prevalence of isthmic spondylolisthesis of 8.2% [2]. Although patients with isthmic spondylolisthesis are usually asymptomatic, in some patients symptoms can develop, such as neurogenic claudication or lumbar radiculopathy. While conservative treatment (i.e. pain management and/or physical therapy) can be helpful, surgical treatment is warranted in those patients refractory to conservative treatment, progressive slippage, and in patients with disabling neurological symptoms [3–5]. 

Surgical management generally consists of nerve root decompression and subsequent spinal fusion. Several spinal fusion procedures have been described, including anterior and posterior approaches. Among these procedures, two frequently performed procedures are posterolateral fusion (PLF) and posterior lumbar interbody fusion (PLIF). In posterolateral fusion, the affected segments are immobilized by implanting bilateral pedicle screws in both segments, which are subsequently connected with rods. In posterior lumbar interbody fusion, additionally the disc is removed, whereafter the intervertebral space is filled with bone grafts or cages. Studies have shown that the latter approach not only improves spinal alignment but also provides anterior column support and increases stiffness of the construct [6–8]. 

Even though both PLF and PLIF are common surgical procedures in the treatment of isthmic spondylolisthesis, considerable evidence comparing the clinical outcomes and quality of life between the two surgical techniques is lacking. Furthermore, radiological outcomes in terms of fusion rate between the two surgical procedures is unclear.

In a recent meta-analysis, Li et al. demonstrated superiority of PLIF compared to PLF in terms of clinical outcome and fusion rate, whilst showing similar surgical outcomes and postoperative complications. Although Li et al. found superior clinical outcomes for PLIF, it is crucial to consider the limitations, which mainly concern the inclusion of patients with degenerative spondylolisthesis, having a substantially distinct pathophysiology. Furthermore, ODI scores were dichotomized, which causes a loss of accuracy in outcome.

The aim of this review is to compare the clinical and radiological outcomes between posterolateral fusion and posterior lumbar interbody fusion in adults with isthmic spondylolisthesis.

## **Materials** and **Methods**

### Protocol and registration

This systematic review was conducted according to the Preferred Reporting Items for Systematic Reviews and Meta-analyses (PRISMA) guidelines. The study protocol is registered under PROSPERO 2022 CRD42022338983 (Available from: https://www.crd.york.ac.uk/prospero/display_record.php?ID=CRD42022338983).

### Eligibility criteria

Studies were included comparing clinical outcomes of posterolateral fusion (PLF) versus posterior lumbar interbody fusion in patients with isthmic spondylolisthesis. Both retrospective and prospective studies were eligible for inclusion. The inclusion criteria were (1) randomized controlled trials and cohort studies, (2) written in English or Dutch, (3) published after the date 01-01-2000, (4) measuring functional outcome at a minimum of 2 time points with a minimum postoperative follow-up of 2 months, comparing PLF with PLIF, (5) with a minimum of 20 participants. Exclusion criteria were (1) patient populations younger than 18 years old or older than 80 years old, (2) studies comparing minimally invasive interventions, (3) studies with cases of isthmic spondylolisthesis.

### Information sources and search

Potentially relevant articles were searched in the available literature through a predetermined search strategy, through consultation of a medical librarian. Various databases were included in the search, consisting of PubMed, Embase, Web of Science, COCHRANE Library and Emcare. MEDLINE was excluded as a database, because records identified in MEDLINE are also included in Pubmed. For each database, the search strategy was adjusted to enable proper search results. All searches were performed on March 28, 2023. The guiding search strategy can be found in the Supplementary Appendix.

### Study selection

Two independent reviewers (KA and SB) assessed the retrieved abstracts obtained through the search strategy. Firstly, the articles were selected by assessing the title and abstract for eligibility according to the inclusion and exclusion criteria (abstract screening). Studies that could not be excluded solely on basis of the title or abstract were included until proven otherwise. Secondly, full texts were retrieved from the selected abstracts, to evaluate eligibility based on the full text (full text screening). If a consensus between KA and SB could not be reached during both abstract screening and full text screening, a third reviewer was consulted (CVL) to resolve the discrepancy by discussion.

### Data collection process

The following variables were extracted from the included articles: study data (e.g., author, publishing year, study design, and inclusion dates), baseline study characteristics (e.g., number of participants, mean/median age, and Meyerding grading), mean follow-up (FU), primary and secondary outcome measures on various follow-up times, surgical outcomes (e.g., blood loss, surgery time, postoperative complications), and reoperation incidence.

### Risk of bias in individual studies

Studies were assessed for their risk of bias. The Newcastle Ottowa Scale was used to assess risk of bias of cohort studies, whereas the modified Cochrane Collaboration Tool was used to assess risk of bias in randomized clinical trials. The risk of bias assessment tools can be found in the Supplementary Appendix. Both reviewers (KA and SB) performed a risk of bias assessment of the included studies. In case a consensus could not be reached, a third reviewer (CVL) was involved for discussion. Studies were categorized in three groups according to the risk of bias (low– moderate– high).

### Statistical analysis

Descriptive data from each individual study were summarized on the predefined variables. All meta-analyses were performed with the Review Manager software (RevMan version 5.4; The Nordic Cochrane Center, The Cochrane Collaboration, Copenhagen, Denmark) [9]. The chi-squared (*x*^*2*^) test was used to assess heterogeneity in the forest plots, and expressed by calculating the *I*^*2*^ statistic. The DerSimonian- and Laird method was used to calculate the heterogeneity variance τ^2^, and p values lower less than 0.05 were considered significant.

Sensitivity analyses and subgroup analyses were performed as necessary.

## Results

### Study selection

**Fig. 1:**
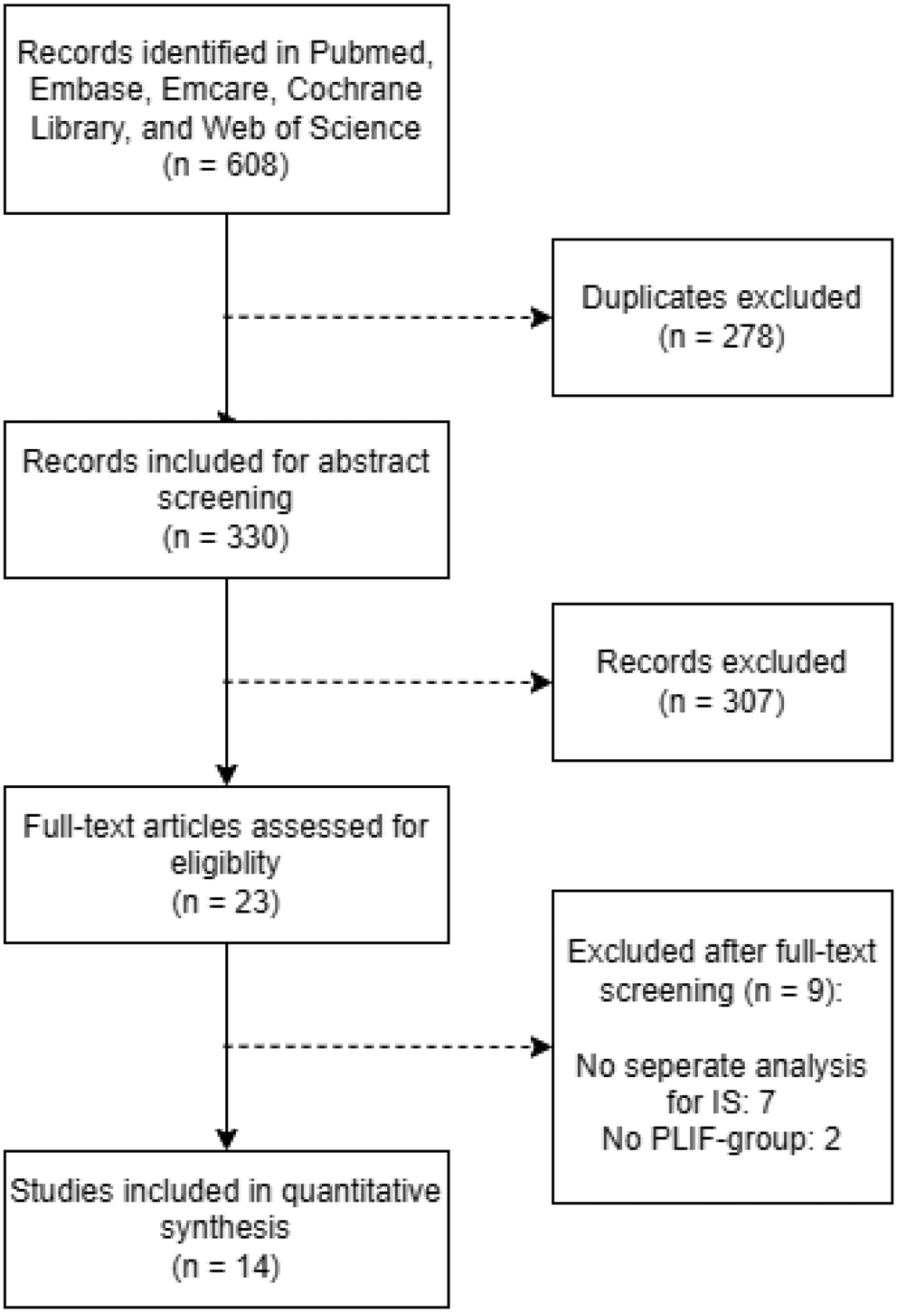
Study inclusion flow chart

The summary of selected relevant studies is summarised in Fig. 1. Our search yielded 608 studies from PubMed, Embase, Emcare, Cochrane Library, and Web of Science. After removing duplicates, 330 studies remained and were eligible for abstract screening. Of these 330 studies, 23 studies were eligible for full-text screening. Nine out of 23 studies were eventually excluded. These excluded studies consisted of 7 studies that had no subgroup analysis for isthmic spondylolisthesis, and two studies that had no study participants treated with PLIF. Finally, 14 studies were included in this systematic review.

### Study characteristics

Among the 14 studies, 4 were RCTs, 6 were retrospective cohorts, and 4 were prospective cohorts of which 2 with historical control. The mean follow-up ranged from 12 to 132 months. In total, there were 1941 participants included, consisting of 1039 (54%) receiving PLF and 902 (46%) participants receiving PLIF. The study by Endler et al. split the PLF group into two: instrumented PLF and non-instrumented PLF [10]. The study by Chan et al. combined transforaminal (TLIF) and posterior lumbar interbody fusion in the PLIF group without specifying results for TLIF and PLIF separately [11]. The characteristics of the included studies are shown in Table 1.

**Table 1:** Study characteristics

Study	Design	Research period	Surgical types	Number of participants PLF/PLIF		Average age PLF/PLIF	Meyerding grade	Follow-up (months)	Functional outcomes	Quality of Life outcomes
Barbanti Bròdano et al. 2010 [14]	Retrospective cohort	2003–2005	PLF and PLIF		43/28	51.6 (8.6)/54.8 (8.6)	I and II	62.1 (51–78)	ODI, RMDQ, VAS	-
Chan et al. 2020 [11]	Retrospective cohort	2013–2017			48/252	43.7/50.1	I-V	12	ODI, VAS	PCS12
Cunningham et al. 2013 [15]	Prospective cohort	1997–2006	PLF and PLIF		21/31	46 (21–70)/43 (21–72)	I and II	94 (76–121)	RMDQ, LBOS	SF-6D, SF-12v2
Dehoux et al. 2004 [17]	Prospective cohort	NS	PLF and PLIF		25/27	42.4 (14–63)/39.5 (14–56)	I- III	(75–100)	Beaujon score	-
Ekman et al. 2007 [16]	Prospective cohort with historical control	1997–2003	PLF and PLIF		77/86	39/40	I- III	24	ODI, DRI	-
Endler et al. 2019 [10]	Prospective cohort with historical control	1997–2003	PLF* and PLIF		71/73	39 (37–42)/40 (37–42)	I- III	132 (60–192)	ODI, DRI	SF-6D mental, SF-6D physical
Endler et al. 2017 [24]	Retrospective cohort	1999–2008	PLF and PLIF		102/452/211	42 (40–44)/43 (42–44)/42 (41–44)	NS	82.8	ODI, VAS	SF-36, EQ-5D
La Rosa et al. 2003 [25]	Retrospective cohort	1997–2000	PLF and PLIF		18/17	57.2 (32–74)	I-III	24	Prolo classificiation	-
Kaliya et al. 2020 [26]	Retrospective cohort	2003–2010	PLF and PLIF		37/27	46.3 (16.4)/48.7 (13)	I-II	24	ODI, VAS	-
Madan et al. 2002 [27]	Retrospective cohort	1995–1998	PLF and PLIF		21/23	42.2 (28–66)/41.15 (24–67)	I and II	35 (25–60)	ODI, MSPQ + ZDS	-
Abdelkader et al. 2019 [28]	RCT	NS	PLF and PLIF		20/20	44.1 (7.34)/44.15 (6.9)	I and II	24	JOA, VAS	ADL
Farrokhi et al. 2012 [13]	RCT	2008–2010	PLF and PLIF		40/40	NS	NS	12	ODI	-
Lee et al. 2014 [29]	RCT	2012	PLF and PLIF		39/42	53.4 (2.3)/53.7 (2.1)	I, II and III	24	ODI, VAS	-
Müslüman et al. 2011 [12]	RCT	2001–2007	PLF and PLIF		25/25	47.3/50.6	I and II	40 (18–72)	ODI, VAS	SF-36

PLF = Posterolateral Fusion, PLIF = Posterior Lateral Interbody Fusion, ODI = Oswestry Disability index, RMDQ = Roland Morris Disability Questionnaire JOA = Japanese Orthopedic Association score, SF-36 = Short Form Health survey, * = Endler et al. 2019 made a distinction between instrumented and noninstrumented PLF

### Risk of bias within studies

One of the RCTs had an overall low risk of bias, two RCTs had a moderate risk of bias and 1 RCT had a high risk of bias. Five of the cohort studies had an overall low risk of bias, and 5 studies had a high risk of bias. The scoring of the individual RCTs is summarized in Table 2 and the scoring of the individual cohort studies is summarised in Table 3.

**Table 2:** Risk of bias randomized controlled trials

Study	Randomization	Allocation concealment	Blinding of participants and personnel	Blinding of outcome assessor	Follow-up	Selective reporting	Overall risk of bias
Abdelkader et al. 2019	High	High	High	High	Low	Low	High
Farrokhi et al. 2012	Low	Low	High	Low	Low	Low	Low
Lee et al. 2014	Low	Low	High	high	Low	Low	Moderate
Müslüman et al. 2011	Low	Low	High	High	Low	Low	Moderate

High = high risk of bias. Low = low risk of bias. Moderate = moderate risk of bias

**Table 3:** Risk of bias cohort studies

Study	Selection	Outcome	Follow-up	Total score
Barbanti Bròdano et al. 2010	4	1	0	5 (high)
Chan et al. 2020	4	1	0	5 (high)
Cunningham et al. 2013	4	1	0	5 (high)
Dehoux et al. 2004	4	1	2	7 (Low)
Ekman et al. 2007	4	1	2	7 (low)
Endler et al. 2019	4	1	0	5 (high)
Endler et al. 2017	4	1	2	7 (low)
La Rosa et al. 2003	4	1	0	5 (high)
Kaliya-Perumal et al. 2020	4	1	2	7 (low)
Madan et al. 2002	4	1	2	7 (Low)

A maximum of 4 points can be rewarded for ‘Selection”, 1 point for ‘Outcome’ and 2 points for ‘Follow-up’. A maximum of 7 points in total can be rewarded to a study. High = high risk of bias. Low = low risk of bias. Moderate = moderate risk of bias

### Clinical outcome

**Fig. 2:**
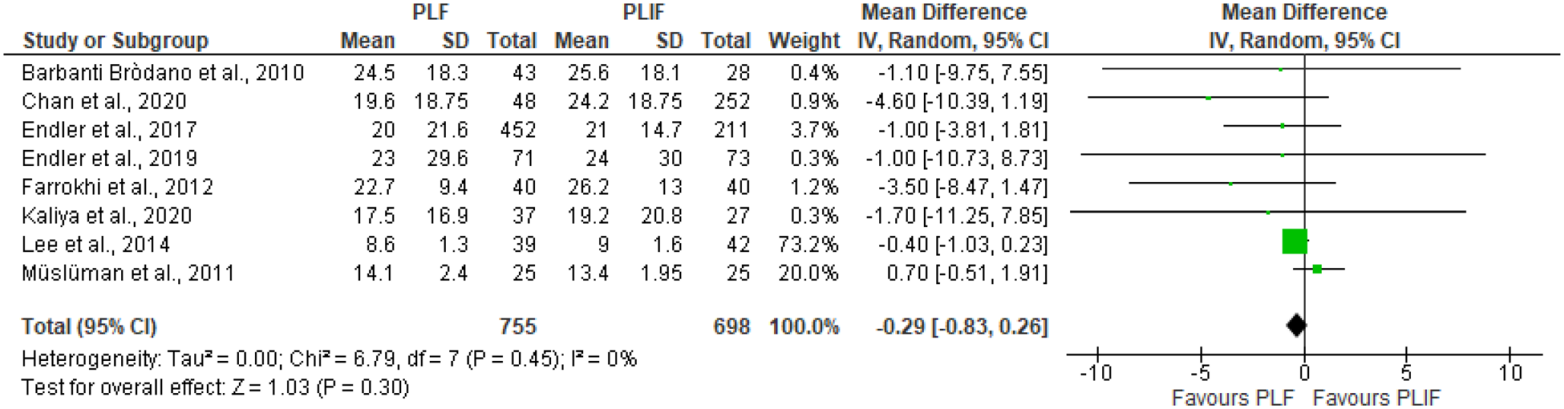
Forest plot of included studies, expressed in ODI scores

**Fig. 3:**
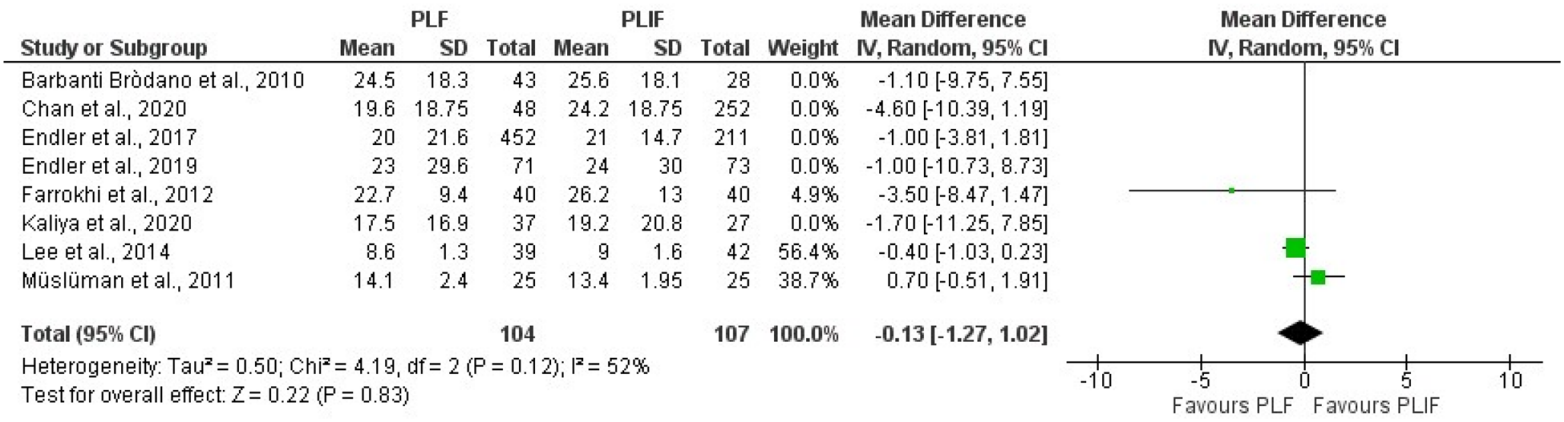
Forest plot of sensitivity analysis in ODI scores, with only RCT’s included

**Fig. 4:**
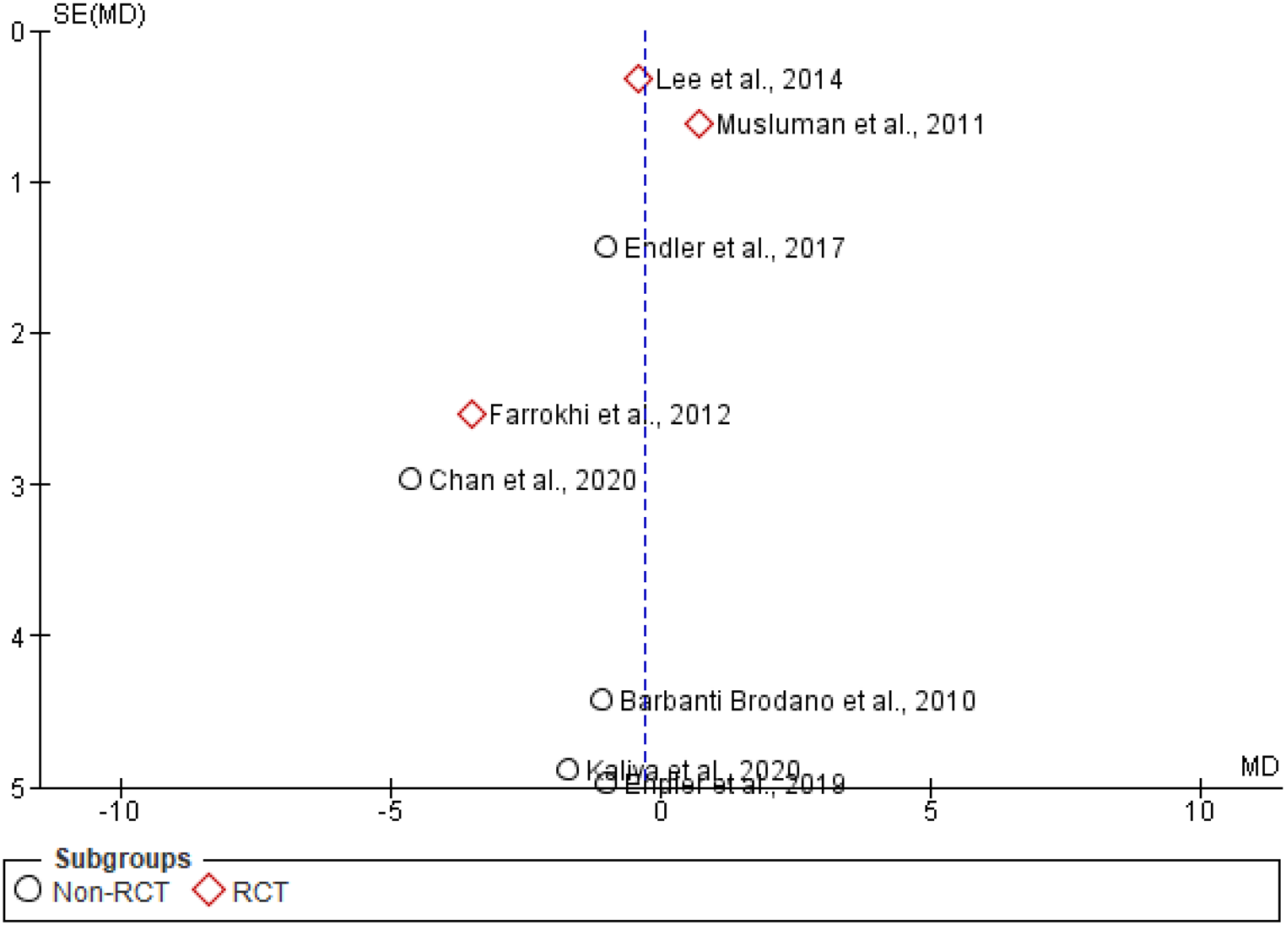
Funnel plot of ODI scores with RCTs identified as subgroup

Ten studies used the ODI to assess clinical outcome, three of which were RCTs. Two RCTs yielded a significant difference in favour of PLIF [12, 13]Farrokhi et al. (low risk of bias) evaluated outcome at 12 months FU in 80 patients, resulting in a significant circa 20 points difference in ODI between PLIF and PLF [12], which can be considered as exceeding the MCID of 15 and thus clinically relevant [14]. Müslüman et al. (moderate risk of bias) only demonstrated a significant, but not clinically relevant, 5-point difference at 3 months in favour of PLIF, that disappeared at the 40-month evaluation point [13]. The third RCT by Lee et al. (moderate risk of bias) only demonstrated a 0.4 non-significant difference between the 81 patients in the two treatment arms [15]. Pooling the ODI results of all studies yielded no differences between both surgical approaches, with an estimated pooled mean difference of -0.29 (95% CI [-0.83, 0.26], *P* = 0.45) (Fig. 2). Both subgroup analyses and sensitivity analyses including only RCTs showed a smaller effect size for RCTs, with an estimated pooled mean difference of -0.13 (95% CI [-1.27, 1.02], *P* = 0.83). There was no difference in effect size between the subgroups (*P* = 0.26). However, this analysis was highly heterogeneous, with an I^2^ of 52% (Fig. 3, Supplementary Fig. 1). Visual inspection of the funnel plot showed minor asymmetry (Fig. 4).

Only two articles, Barbanti Bròdano et al. (high risk of bias) and Cunningham et al. (high risk of bias), evaluated the Roland Disability Score (RMDQ) and did not observe a significant difference [16, 17] One study, Abdelkader et al. (high risk of bias), assessed the adjusted JOA score and they did not establish a significant difference. Three studies used the SF-36, of which only Müslüman et al. retrieved a significant difference at 3 and 40 months in favour of PLIF [13]. 

Seven studies reported the postoperative VAS scores for leg and back pain. Only one study, Müslüman et al., yielded a significant difference in favour of PLIF for the VAS back pain score at 3 (12 mm) and 40 (6 mm) months postoperative. No significant differences were demonstrated for the VAS leg pain scores. All clinical outcomes are displayed in Tables 4 and 5.

**Table 4:** Results of each included study (functional outcomes)

Study	Type of surgery	Follow-up (months)	ODI	RMDQ	VAS leg	VAS back	Other
Barbanti Bròdano et al. 2010	PLF	62	24.5 (18.3)*	6.9 (6.3)*	3.5 (3.2)*	3.8 (2.7)*	
	PLIF	62	25.6 (18.1)*	7.2 (6.2)*	4.1 (2.8)*	3.0 (2.0)*	
Chan et al. 2020	PLF	12	19.6*		2.25*	3.38*	
	PLIF	12	24.21*		2.97*	3.21*	
Cunningham et al. 2013	PLF	12		6.7 (6.8)*			LOBS: 39.7 (18.5)*
	PLIF	12		5.3 (5.9)*			LOBS: 52.3 (15.3)*
	PLF	94					
	PLIF	94					
Dehoux et al. 2004	PLF						
	PLIF						
Ekman et al. 2007	PLF	12					35* DRI: 31*
	PLIF	12					35* DRI: 30*
	PLF	24	25				37* DRI: 29*
	PLIF	24	25				35* DRI: 30*
Endler et al. 2019	PLF	132	23 (17–30)*				Pain Index: 28 (19–36)*
	PLIF	132	24 (17–31)*				Pain Index: 36 (28–44)*
Endler et al. 2017	PLF	12	21 (19–23)*		20 (17–23)*	27 (24–29)*	
	PLIF	12	22 (19–24)*		21 (17–25)*	27 (23–31)*	
	PLF	24	20 (18–22)*		22 (19–24)*	26 (24–29)*	
	PLIF	24	21 (18–23)*		23 (19–27)*	27 (23–30)*	
	PLF	60	20(18–23)*		23 (20–26)*	28 (25–31)*	
	PLIF	60	22 (18–25)*		23 (18–27)*	27 (22–31)*	
La Rosa et al. 2003	PLF	24					motor deficit: 0* sensory deficit: 2* economic score: 3.8* functional score: 3.8*
	PLIF	24					motor deficit: 1* sensory deficit: 1* economic score: 3.8* functional score: 4.2*
Kaliya-Perumal et al. 2020	PLF	24	17.5 (16.9)*		1.8 (1.9)*	1.9 (1.6)*	
	PLIF	24	19.2 (20.8)*		1.2 (1.6)*	1.7 (2.1)*	
Madan et al. 2002	PLF	25	24.54 (4–68)*				MSPQ + ZDS: 21 (0–49)*
	PLIF	25	38.34 (2–86)*				MSPQ + ZDS: 21 (0–49)*
Abdelkader et al. 2019	PLF	24			2.85 (0.37)*	2.2 (0.6)*	JOA: 23.9 (1.9)*
	PLIF	24			2.7 (0.47)*	2.05 (0.6)*	JOA: 25.15 (1.4)*
Farrokhi et al. 2012 [13]	PLF	12	51**				
	PLIF	12	33.11**				
Lee et al. 2014	PLF	24	8.6 (1.3)*		0.9 (0.3)*	1.5 (1.2)*	
	PLIF	24	9.0 (1.6)*		1.0 (0.4)*	1.6 (1.0)*	
Müslüman et al. 2011	PLF	3	18.20 (3.65)**		1.2 (1.04)*	2.32 (0.9)**	
	PLIF	3	13.60 (1.95)**		1.2 (0.74)*	1.12 (0.66)**	
	PLF	40	14.12 (2.42)*		1.08 (0.9)*	1.8 (0.57)**	
	PLIF	40	13.40 (1.95)*		1.0 (0.64)*	1.2 (0.57)**	
	PLIF						

Dehoux et al. presented their results in figures only. * = significant result compared to baseline. ** = significant result compared to the other surgical intervention. PLF = Posterolateral Fusion, PLIF = Posterior Lateral Interbody Fusion, ODI = Oswestry Disability index, RMDQ = Roland Morris Disability Questionnaire JOA = Japanese Orthopedic Association score, SF-36 = Short Form Health survey, VAS = Visual Analog Scale, EQ-5D = EuroQol 5D, ADL =

**Table 5:** Results of each included study (quality of life outcomes)

Study	Type of surgery	Follow-up (months)	PCS12	SF-6D R2	EQ-5D	SF-12v2	SF-6D mental	SF-6D physical	SF-36 MCS	SF-36 PCS	ADL score	SF-36
Barbanti Bròdano et al. 2010	PLF	62										
	PLIF	62										
Chan et al. 2020	PLF	12	11.68*									
	PLIF	12	10.88*									
Cunningham et al. 2013	PLF	12										
	PLIF	12										
	PLF	94		0.653 (0.581–0.870)		39.7 (28.2–51.6)**						
	PLIF	94		0.863 (0.660–0.922)		48.7 (37.8–57.8)**						
Endler et al. 2019	PLF	132					44 (40–49)*	40 (36–45)*				
	PLIF	132					48 (43–52)*	39 (35–44)*				
Endler et al. 2017	PLF	12			0.69 (0.66–0.72)*				44 (43–45)*	46 (44–47)*		
	PLIF	12			0.67 (0.62–0.71)*				44 (42–46)*	44 (42–46)*		
	PLF	24			0.71 (0.68–0.74)*				44 (43–45)*	46 (45–47)*		
	PLIF	24			0.69 (0.65–0.73)*				43 (41–45)*	46 (44–47)*		
	PLF	60			0.70 (0.66–0.73)*				43 (42–44)*	47 (46–48)*		
	PLIF	60			0.68 (0.63–0.74)*				44 (42–46)*	47 (45–49)*		
Abdelkader et al. 2019	PLF	24									10.2 (1.2)*	
	PLIF	24									12 (1.03)*	
Farrokhi et al. 2012	PLF	12										
	PLIF	12										
Lee et al. 2014	PLF	24										
	PLIF	24										
Müslüman et al. 2011	PLF	3										75.24 (6.02)**
	PLIF	3										80.20 (5.75)**
	PLF	40										81.48 (6.83)**
	PLIF	40										85.88 (5.6)**
	PLIF											

Dehoux et al. presented their results in figures only. * = significant result compared to baseline. ** = significant result compared to the other surgical intervention. PLF = Posterolateral Fusion, PLIF = Posterior Lateral Interbody Fusion, ODI = Oswestry Disability index, RMDQ = Roland Morris Disability Questionnaire JOA = Japanese Orthopedic Association score, SF-36 = Short Form Health survey, VAS = Visual Analog Scale, EQ-5D = EuroQol 5D, OLBP = Oswestry low back pain disability scale, ADL =

### Fusion rate

Six studies compared fusion rates between PLF and PLIF treatment. Only two of these studies yielded a significant difference [12, 13]. In the study by Farrokhi et al., the fusion rate was classified as “good” or “fair + bad”. The percentages that were rated “good” at 12 months in the PLF group and the PLIF group were 66.7% and 89.1%, respectively (*p* = 0.012) [12]. In the study of Müslüman et al. there was a significant difference at 6, 12 and 24 months in favour of PLIF (*p* < 0.005) [13].

### Postoperative complications

Nine studies reported on postoperative complications (Table 6). Six studies did not find a significant difference in complications between PLF and PLIF, one study reported more complications in the PLIF group [18], and three studies reported more complications in the PLF group [10, 13, 19]. The study by Ekman et al. (low grade of bias) documented 12 complications in the PLIF group (wound infection (*n* = 3), persisting leg pain (*n* = 4), neurological deficit (*n* = 3), thrombo-embolic events (*n* = 2), ), and 4 complications in the PLF group (persisting leg pain (*n* = 1), neurological deficit (*n* = 2), unilateral blindness (*n* = 1) [18]. Dehoux et al. (low risk of bias), reported more complications in the PLF group (*n* = 11) of which 8 resulted in hardware removal, and only 2 complications in the PLIF group, in which 1 required removal of hardware [19] Endler et al. (high risk of bias), reported more reoperations in the PLF group (*n* = 11: due to nerve root irritation (*n* = 2), pseudoarthrosis (*n* = 1), disc herniation (*n* = 1), and back pain (*n* = 7)). In the PLIF group, 6 patients were reoperated (pseudoarthrosis (*n* = 2), disc herniation (*n* = 1), and backpain (*n* = 3) [10]The third study that demonstrated more complications in the PLF group is Müslüman et al., reporting 10 complications in the PLF group (4 of those concerned nonunion), and 3 complications in the PLIF group [13].

Six studies stated the reoperation rate in their article, five of them reported that the rate was not significantly different and 1 article did not comment on significance.

**Fig. 5:**
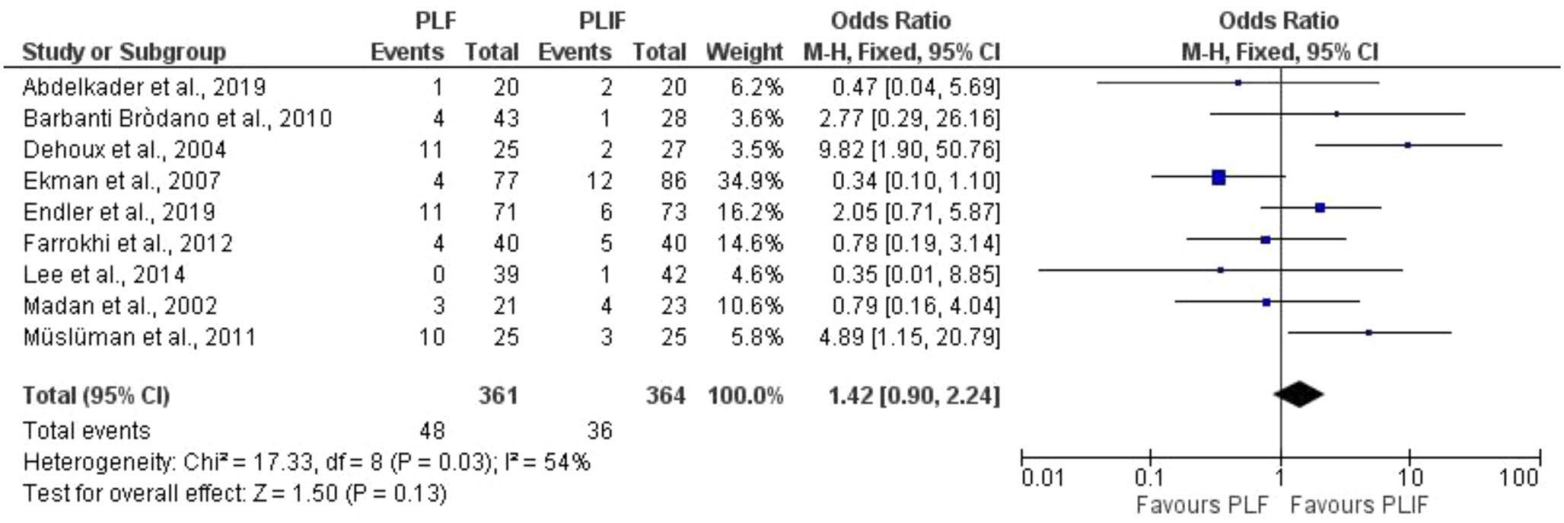
Forest plot of complications included studies

**Fig. 6:**
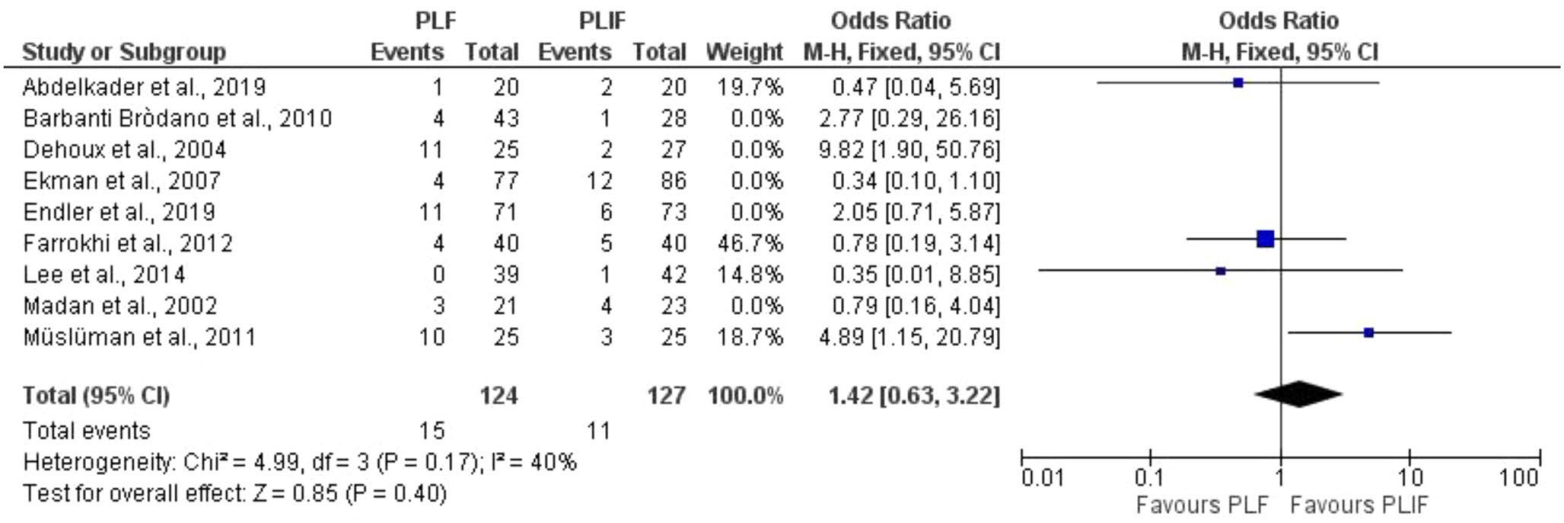
Forest plot of sensitivity analysis in complications, with only RCT’s included

Pooling the complication rate in a forest plot yielded no difference between the treatments (estimated pooled odds ratio 1.42 (95% CI [0.90, 2.24], *P* = 0.13), I^2^ = 54%; Fig. 5). When performing a sensitivity analysis by only including RCTs, likewise, no difference between both treatments was demonstrated (estimated pooled odds ratio 1.42 (95% CI [0.63, 3.22], *P* = 0.40), I^2^ = 0.40 (Fig. 6). Upon subgroup analyses based on study design, the heterogeneity was reduced to 40% in the RCT studies, suggesting that study design contributed to the variability. However, no differences in effect sizes were found between RCTs and non-RCTs (*P* = 1.00) (Supplementary Fig. 2).

### Surgical outcomes

**Fig. 7:**
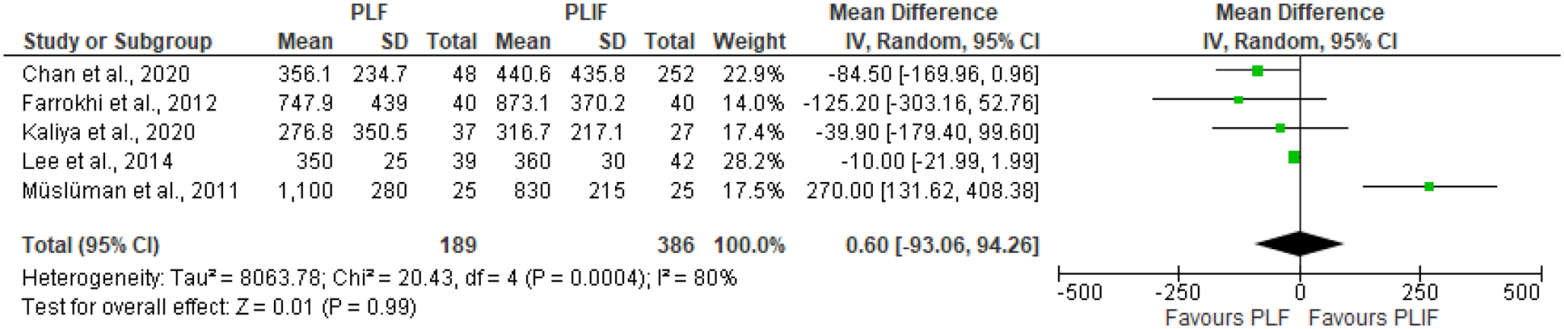
Forest plot of perioperative blood loss

Six studies investigated perioperative blood loss. Farrokhi et al. observed greater blood loss in the PLIF group, while Müslüman et al. reported significantly higher blood loss in the PLF group. The results from Abdelkader et al. were excluded from the forest plot due to incomplete statistical data. The pooled mean difference in perioperative blood loss, derived from five studies, was comparable in both PLF and PLIF group, yielding a mean difference of 0.60 (95% CI [-93,06, 94,26], *P* = 0.99, I^2^ = 80) (Table 6; Fig. 7).

**Fig. 8:**
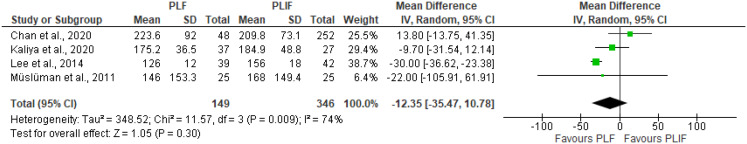
Forest plot of surgery time

Surgery time was reported by five studies, with four studies providing statistical results. Only Lee et al. found a significant difference in surgery time in favour of PLF (126 (± 12) minutes versus 156 (± 18) minutes in the PLF and PLIF group respectively, *P* = 0.03). A forest plot indicated no overall difference between both groups in terms of surgery time, with a pooled mean difference of -12.35 (95% CI [-35.47, 10.78], *P* = 0.30, I^2^ = 74%) (Table 6; Fig. 8).

**Table 6:** Surgical characteristics and complications

Study	Type of surgery	Blood loss	Operation time	Fusion rate	Complications
Barbanti Bròdano et al. 2010	PLF			95%	4 (9.3%): revision surgery of which 2 for pseudoarthrosis
	PLIF			97%	1 (3.6%): revision surgery for pseudoarthrosis
Chan et al. 2020	PLF	356.1 (234.7)	223.6 (92.0)		
	PLIF	440.6 (435.8)	209.8 (73.1)		
Cunningham et al. 2013	PLF				
	PLIF				
Dehoux et al. 2004	PLF			68%	11: 1 epidura haematoma, 8 hardware removal, 2 disc herniation
	PLIF			93%	2: 1 sexual impotence with bladder dysfunction (normal function at follow-up), 1 mechanical failure
Ekman et al. 2007	PLF				**4: 2 permanent L5 injuries, 1 permanent unilateral blindness, 1 transient dermatomal pain
	PLIF				**12: 3 deep wound infection, 2 permanent leg pain, 2 transient leg pain, 1 permanent foot drop, 1 transient foot drop, 1 deep vein thrombosis, 1 pulmonary embolus, 1 postoperative paraparesis
Endler et al. 2019	PLF				11: 2 reoperations due to nerve root irritation, 1 pseudoarthrosis, 1 due to disc herniation, 7 implant removals due to local irritation
	PLIF				6: 2 reoperations due to pseudoarthrosis, 1 due to disc herniation, 3 implant removals due to irritation
Endler et al. 2017	PLF				
	PLIF				
La Rosa et al. 2003	PLF			88.9%	0
	PLIF			100%	0
Kaliya-Perumal et al. 2020	PLF	276.8 (350.5)	175.2 (36.5)		
	PLIF	316.7 (217.1)	184.9 (48.8)		
Madan et al. 2002	PLF				3: 3 losses of correction
	PLIF				4: 1 superficial infection, 1 reoperation for hardware failure, 2 nonunions
Abdelkader et al. 2019	PLF	1000	95		1: 1 infection of iliac graft site
	PLIF	1100	105		2: 2 transient foot drop
Farrokhi et al. 2012	PLF	747.87 (439)**		66.7%	4.3% cerebrospinal fluid leakage, 2.1% infection, 4.3% permanent motor impairement
	PLIF	873.07 (370.24)**		89.1%	5.3% cerebrospinal fluid leakage, 2.5% infection, 5% permanent motor impairement
Lee et al. 2014	PLF	350 (25)	126 (12)**	84.6%	0
	PLIF	360 (30)	156 (18)**	85.7%	1: 1 deep infection
Müslüman et al. 2011	PLF	1100 (280)**	146 (105–300)	6 months: 72%**, 1 year: 80%**, 2 years: 84%**	10: 1 transient nerve palsy, 2 deep infection, 3 pain in bone graft donor site, 4 nonunion
	PLIF	830 (215)**	168 (120–310)	6 months: 84%**, 1 year: 96%**, 2 years: 100%**	3: 1 transient nerve palsy, 1 deep infection, 1 cage dislocation

** = significant result compared to the other surgical intervention

## Discussion

### Summary of evidence

This systematic review included a fair number of studies, but still it remains undetermined whether PLF is as effective as PLIF in functional outcome, fusion rate, and whether the complication rate is comparable. Two RCTs yielded a significantly better functional outcome in PLIF, though only one demonstrated clinical relevance, and pooling the available data could not indicate a difference. In addition, the sensitivity analysis that was performed on RCTs, to assess the robustness of the meta-analysis pooling, revealed no significant differences in outcome. Fusion rate was reported to be significantly better in PLIF in two studies, but the other four studies could not confirm this. And finally, after pooling the complication data, no difference could be demonstrated between the two types of interventions either. Based on this overview of data, there is not enough evidence to choose either PLF or PLIF as the best treatment option for adults with isthmic spondylolisthesis. In addition, none of the articles corrected for possibly confounding factors, consolidating the conclusion that data available in current literature cannot reveal a difference between the two interventions.

### Disc height is one of the possibly confounding factors

In the surgical treatment of isthmic spondylolisthesis, a commonly assumed notion is that adding interbody fusion leads to more anterior column support, indirect foraminal decompression and restoration of spinal sagittal alignment. Considering most studies being non-randomized and retrospective, the question is raised as to why surgeons choose PLF for some patients and PLIF for other patients. Disc height was described to play an important role in choosing for PLF of PLIF. La Rosa et al. discussed a theoretic possibility that the narrowed disc height between end plates can biomechanically contribute to stability and that therefore the need for interbody fusion is lacking [20] They conclude that this explains the satisfactory outcomes of PLF in terms of back pain and fusion rate. Future studies should include disc height and general lumbar spine degeneration as factors to be taken into consideration when comparing the success of both interventions.

Furthermore, the degree of slippage is a factor of importance and should also be considered a possibly confounding factor. Abdelkader et al. stated that PLIF may be better for high grade spondylolisthesis and PLF sufficient for low grade spondylolisthesis [21] Their line of reasoning is that PLIF is associated with longer operation time, more blood loss and higher risk of postoperative radicular pain and drop foot, and they claim that PLF is less demanding and has relatively less risks, and thus more suitable for low grade spondylolisthesis. This is one of the assumptions that can be rejected after the evaluation of complications and surgical characteristics, such as surgery time and perioperative blood loss in the current review: PLIF was not associated with a higher complication rate. Kaliya et al. were even more convinced that PLIF should be performed in a higher grade of slippage [22]. They excluded these patients from their study and offered them PLIF regardless of other factors.

The results of this meta-analysis show no statistically significant difference between the two surgical interventions. However, these findings should be interpreted with caution. In contrast to posterolateral fusion, posterior lumbar interbody fusion involves a discectomy and the posterior placement of an interbody, restoring the segmental height and thereby leading to an indirect decompression of the neural foramina. While this technique theoretically adds segmental stability, it also carries specific interbody fusion associated risks. During placement of the interbody cage, the superior exciting nerve root could be injured. This is especially important in patients with a history of previous spinal surgery at the affected segments, where fibrous tissue development can complicate the identification of the nerve root. Although PLIF is generally considered a more invasive surgical procedure, the complication rate reported in this meta-analysis was compared to PLF. While subgroup analyses yielded similar estimated pooled effect for complication rate, caution is warranted, as nerve root injury is a relatively rare complication, and the included studies have a relatively small sample size and may be underpowered to detect a clinically relevant difference. Moreover, some of the included studies were retrospective and thus prone to patient selection bias. Prospective study designs with clear and uniform inclusion- and exclusion criteria are essential and can mitigate this bias, making the comparison in complication rate more valid and practical.

Similarly, this meta-analysis found no difference in surgery time and perioperative blood loss between PLF and PLIF, challenging the conventional belief that PLIF is associated with higher blood loss and longer operative times due to its more invasive nature. Surgical factors were not reported in detail by the included articles, and variations in perioperative management, such as the use of hemostatic agents, intraoperative blood salvage methods, bone grafting methods, and advancements in instrumentation, may have affected these results. The combination of these factors could account for the comparable outcomes in terms of blood loss and surgery time between the two surgical procedures. While most studies included in the meta-analysis show a trend towards increased blood loss and longer surgery times with PLIF, the data remains heterogeneous and should be interpreted with caution. Consequently, in their decision-making process, clinicians should incorporate surgery time and blood loss as part of a broader, patient-specific evaluation. Another factor of importance is age. Older patients generally have a more degenerated spine with a narrowed disc space, and they have osteophytes consolidating the vertebra. If reducing the facet joint satisfactorily decompresses the spinal nerve root, no additional benefit of interbody fusion is presumed to be present. Moreover, the elderly are usually more vulnerable to complications. Other possibly confounding factors that may be important are the condition of the facet joint and the factors that influence fusion, like smoking or bone mass density and structure [23–26]. 

In order to correct for these confounding factors, an RCT could be conducted. It is unfortunate that the four RCTs described in literature do not elaborate on these possible confounding factors. With the current knowledge however, a design of a future RCT to find a proper answer to the question whether PLF and PLIF are comparable would be ethically challenging or nearly unethical due to the significant role that these speculative factors play in the clinical decision-making of treating isthmic spondylolisthesis. It would be more realistic to conduct an observational cohort study and to carefully register the mentioned confounders.

Several previously reported reviews have evaluated the clinical outcomes of PLF and PLIF for symptomatic isthmic spondylolisthesis. However, the heterogeneity of included patient populations hinders drawing firm conclusions and encouraged us to write the current review. A specific shortcoming in previous reviews is that they not only included cases of isthmic spondylolisthesis but also of degenerative spondylolisthesis [27–30] A systematic review by Jacobs et al. reviewed only older studies, published between 1996 and 2004, and reported a superior clinical outcome of PLF (60–98% excellent) over PLIF (45% excellent) in low-grade isthmic spondylolisthesis [31]. This conclusion differs from ours, but can be explained by the considerable advancements in hardware and surgical techniques that occurred over the years, making interbody fusion less time-consuming and less prone for complications.

Other reviews demonstrate results that are in line with the conclusions in the current review [32–34], but data were not pooled and conclusions were thus less robust.

### Limitations

Unfortunately, there are some limitations that affect this systematic review. Most of the included studies did not randomize patients between treatments, which led to risk of bias, being mainly selection bias illustrated by differences in baseline characteristics. Although the funnel plot demonstrated minor asymmetry, which could raise concerns about possible publication bias, its interpretation and statistical power is limited by the relatively small number of studies included. Moreover, a key limitation was the moderate-to-high heterogeneity that was observed in the pooled results of functional outcome in the RCT studies, complications, and surgical outcomes, indicating that variability within study results was present. While subgroup analyses by study design reduced the heterogeneity for complications in the included RCTs, this could not fully explain the heterogeneity of the data. One plausible source for heterogeneity could be differences in follow-up times, ranging between 12 and 132 months for the included studies; we have used the latest follow-up time, in an attempt to demonstrate the most valid results. Imbalances in sizes of PLF and PLIF groups could also be an explanation for heterogeneity, as was seen particularly with the study of Endler et al. and Chan et al. Other clinical differences within studies, such as age, indications for surgery, and method of fusion, could also explain the heterogeneity, which could not be fully captured in subgroup analyses. Another limitation was the variety of disability questionnaires used to evaluate functional outcome, making comparison between the studies challenging. Furthermore, the patient populations of the different studies are not homogeneous, and even the surgical interventions were not always comparable as in offering uninstrumented fusion as opposed to instrumented, and combining TLIF and PLIF in the interbody fusion group. Grade of slip also differed among the included studies. Barbanti Bròdano et al. included patients with Meyerding I and II [16], but Chan et al. included I, II, III, IV and V [11] Higher-grade spondylolisthesis generally warrants more aggressive stabilization and reduction, which clouds the outcomes in the analysis [11, 16]. Finally, some studies had a very limited number of participants, which makes the results prone to be inconclusive due to small statistical power. In this meta-analysis, a threshold of at least 20 participants per comparative study was set in order to limit the disproportionate effects that smaller studies could have on the outcomes of the meta-analysis. Setting a minimum sample size could ensure the inclusion of more methodologically robust studies, reducing the risk of imprecise effect estimates due to random variation. We deemed a minimum of 20 participants to be necessary to minimize this risk, while preventing the introduction of selection bias that could arise from excluding relevant studies. Nonetheless, the demonstrated heterogeneity of the included studies, best shown in the meta-analysis, which reported a 99% heterogeneity, remains an important limitation of this meta-analysis.

## Conclusions

The outcome data in this review suggest that both PLF and PLIF are satisfactory treatment options for symptomatic isthmic spondylolisthesis. However, the lack of attention for possible confounding factors in the included articles leads to absence of evidence to support either PLF or PLIF as the best treatment option. The results from the RCTs have a tendency to demonstrate a slight preference for the PLIF, but heterogeneity between studies has a negative influence on the interpretation and generalization of results. Future studies should focus on the evaluation of possible confounding factors and clearly defined inclusion and exclusion criteria. This would facilitate comparability and consistency of data, and lead to more robust and reliable conclusions.

## Electronic supplementary material

Below is the link to the electronic supplementary material.

Supplementary Material 1

Supplementary Material 2

## Data Availability

No datasets were generated or analysed during the current study.
