# Supplementary material for: Posterior lumbar fusion with and without interbody fusion in isthmic spondylolisthesis: a systematic review and meta-analysis
Source: Neurosurg Rev. 2025 Jul 28;48(1):581. doi: 10.1007/s10143-025-03703-x (PMC12301273; doi:10.1007/s10143-025-03703-x)
Supplement: Supplementary file 1 — Supplementary Material 1 [file 10143_2025_3703_MOESM1_ESM.docx]

posterior lumbar fusion with and without interbody fusion in isthmic spondylolisthesis: a systematic review and meta-analysis

Kayoumars Azizpour (0000-0002-7520-226X)^1^, M.D., Sverre J. Broekman^1^, B.Sc., Wilco C. Peul^1,2^, M.D., Ph.D., Carmen L.A. Vleggeert-Lankamp^1,2,4^, M.D., M.Sc., Ph.D.

^1^Department of Neurosurgery, Leiden University Medical Center, Leiden, ^2^Department of Neurosurgery, Haaglanden Medical Center, The Hague, ^3^Alrijne Hospital, Leiden & Leiderdorp, ^4^Department of Neurosurgery, Spaarne Hospital, Haarlem/Hoofddorp, the Netherlands

**Corresponding author:**

K. Azizpour, Department of Neurosurgery, Leiden University Medical Center, Albinusdreef 2, 2300 RC Leiden, the Netherlands. 0031 71 5262109, [k.azizpour@lumc.nl](mailto:k.azizpour@lumc.nl)

Search strategy

(("isthmic spondylolisthesis"[tw] OR "isthmic spondylolistheses"[tw] OR (isthmic*[tw] AND ("Spondylolisthesis"[Mesh] OR spondylolisth*[tw])) OR "spondylolytic spondylolisthesis"[tw] OR (spondylolytic*[tw] AND ("Spondylolisthesis"[Mesh] OR spondylolisthes*[tw])) OR "degenerative spondylolisthesis"[tw]  OR (degenerat*[tw] AND ("Spondylolisthesis"[Mesh] OR spondylolisthes*[tw]))) AND ("posterior lumbar interbody fusion"[tw] OR "posterior lumbar interbody fusions"[tw] OR (("posterior"[tw] OR posterior*[tw]) AND ("lumbar"[tw] OR lumba*[tw] OR lumbo*[tw] OR "Lumbar Vertebrae"[Mesh]) AND ("interbody"[tw] OR "inter-body"[tw] OR interbod*[tw]) AND ("fusion"[tw] OR fusion*[tw] OR "Spinal Fusion"[Mesh])) OR "PLIF"[tw] OR "Interbody Fusion"[tw]) AND ("posterolateral fusion"[tw] OR "posterolateral fusions"[tw] OR "postero lateral fusion"[tw] OR "postero lateral lumbar fusion"[tw] OR "posterolateral lumbar fusion"[tw] OR "posterolateral lumbar fusions"[tw] OR "postero lateral interbody fusion"[tw] OR "posterolateral interbody fusion"[tw] OR (("posterolateral"[tw] OR posterolateral*[tw] OR "postero lateral"[tw] OR postero-lateral*[tw]) AND ("fusion"[tw] OR fusion*[tw] OR "Spinal Fusion"[Mesh])) OR "PLF"[tw]))

**NEWCASTLE - OTTAWA QUALITY ASSESSMENT SCALE**

**COHORT STUDIES**

Note: A study can be awarded a maximum of one star for each numbered item within the Selection and Outcome categories. A maximum of two stars can be given for Comparability

**Selection**

1) Representativeness of the exposed cohort

a) truly representative of the average _______________ (describe) in the community **✵**

b) somewhat representative of the average ______________ in the community **✵**

c) selected group of users eg nurses, volunteers

d) no description of the derivation of the cohort

2) Selection of the non exposed cohort

a) drawn from the same community as the exposed cohort **✵**

b) drawn from a different source

c) no description of the derivation of the non exposed cohort

3) Ascertainment of exposure

a) secure record (eg surgical records) **✵**

b) structured interview **✵**

c) written self report

d) no description

4) Demonstration that outcome of interest was not present at start of study

a) yes **✵**

b) no

**Comparability**

1) Comparability of cohorts on the basis of the design or analysis

a) study controls for functional outcomes / pain **✵**

b) study controls for degree of spondylolisthesis (eg Meyerding grading) **✵**

**Outcome**

1) Assessment of outcome

a) independent blind assessment **✵**

b) record linkage **✵**

c) self report

d) no description

2) Was follow-up long enough for outcomes to occur

a) yes (two months follow-up or more) **✵**

b) no

3) Adequacy of follow up of cohorts

a) complete follow up - all subjects accounted for **✵**

b) subjects lost to follow up unlikely to introduce bias - small number lost - > 90 % follow up, or description provided of those lost **✵**

c) follow up rate < 90% and no description of those lost

d) no statement

Revised Cochrane risk-of-bias tool for randomized trials (RoB 2)

TEMPLATE FOR COMPLETION

Edited by Julian PT Higgins, Jelena Savović, Matthew J Page, Jonathan AC Sterne
on behalf of the RoB2 Development Group

**Version of 22 August 2019**

The development of the RoB 2 tool was supported by the MRC Network of Hubs for Trials Methodology Research (MR/L004933/2- N61), with the support of the host MRC ConDuCT-II Hub (Collaboration and innovation for Difficult and Complex randomised controlled Trials In Invasive procedures - MR/K025643/1), by MRC research grant MR/M025209/1, and by a grant from The Cochrane Collaboration.


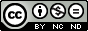


This work is licensed under a [Creative Commons Attribution-NonCommercial-NoDerivatives 4.0 International License](http://creativecommons.org/licenses/by-nc-nd/4.0/).

| **Study details**   \| **Reference** \|  \| \| --- \| --- \|   **Study design**   \| X \| Individually-randomized parallel-group trial \| \| --- \| --- \| \| ⬜ \| Cluster-randomized parallel-group trial \| \| ⬜ \| Individually randomized cross-over (or other matched) trial \|   **For the purposes of this assessment, the interventions being compared are defined as**   \| Experimental: \|  \| Comparator: \|  \| \| --- \| --- \| --- \| --- \|  \| **Specify which outcome is being assessed for risk of bias** \|  \| \| --- \| --- \|  \| **Specify the numerical result being assessed.** In case of multiple alternative analyses being presented, specify the numeric result (e.g. RR = 1.52 (95% CI 0.83 to 2.77) and/or a reference (e.g. to a table, figure or paragraph) that uniquely defines the result being assessed. \|  \| \| --- \| --- \|   **Is the review team’s aim for this result…?**   \| ⬜ \| to assess the effect of *assignment to intervention* (the ‘intention-to-treat’ effect) \| \| --- \| --- \| \| ⬜ \| to assess the effect of *adhering to intervention* (the ‘per-protocol’ effect) \|   **If the aim is to assess the effect of *adhering to intervention***, select the deviations from intended intervention that should be addressed (at least one must be checked):  ⬜ occurrence of non-protocol interventions  ⬜ failures in implementing the intervention that could have affected the outcome  ⬜ non-adherence to their assigned intervention by trial participants  **Which of the following sources were obtained to help inform the risk-of-bias assessment? (tick as many as apply)**  ⬜ Journal article(s) with results of the trial  ⬜ Trial protocol  ⬜ Statistical analysis plan (SAP)  ⬜ Non-commercial trial registry record (e.g. ClinicalTrials.gov record)  ⬜ Company-owned trial registry record (e.g. GSK Clinical Study Register record)  ⬜ “Grey literature” (e.g. unpublished thesis)  ⬜ Conference abstract(s) about the trial  ⬜ Regulatory document (e.g. Clinical Study Report, Drug Approval Package)  ⬜ Research ethics application  ⬜ Grant database summary (e.g. NIH RePORTER or Research Councils UK Gateway to Research)  ⬜ Personal communication with trialist  ⬜ Personal communication with the sponsor |
| --- | --- | --- | --- | --- | --- | --- | --- | --- | --- | --- | --- | --- | --- | --- | --- | --- | --- | --- | --- | --- |

Risk of bias assessment

Responses underlined in green are potential markers for low risk of bias, and responses in red are potential markers for a risk of bias. Where questions relate only to sign posts to other questions, no formatting is used.

**Domain 1: Risk of bias arising from the randomization process**

| **Signalling questions** | **Comments** | **Response options** |
| --- | --- | --- |
| **1.1 Was the allocation sequence random?** |  | Y / PY / PN / N / NI |
| **1.2 Was the allocation sequence concealed until participants were enrolled and assigned to interventions?** |  | Y / PY / PN / N / NI |
| **1.3 Did baseline differences between intervention groups suggest a problem with the randomization process?** |  | Y / PY / PN / N / NI |
| **Risk-of-bias judgement** |  | Low / High / Some concerns |
| Optional: What is the predicted direction of bias arising from the randomization process? |  | NA / Favours experimental / Favours comparator / Towards null /Away from null / Unpredictable |

Domain 2: Risk of bias due to deviations from the intended interventions (*effect of assignment to intervention*)

| **Signalling questions** | **Comments** | **Response options** |
| --- | --- | --- |
| **2.1. Were participants aware of their assigned intervention during the trial?** |  | Y / PY / PN / N / NI |
| **2.2. Were carers and people delivering the interventions aware of participants' assigned intervention during the trial?** |  | Y / PY / PN / N / NI |
| **2.3. If Y/PY/NI to 2.1 or 2.2: Were there deviations from the intended intervention that arose because of the trial context?** |  | NA / Y / PY / PN / N / NI |
| **2.4 If Y/PY to 2.3: Were these deviations likely to have affected the outcome?** |  | NA / Y / PY / PN / N / NI |
| **2.5. If Y/PY/NI to 2.4: Were these deviations from intended intervention balanced between groups?** |  | NA / Y / PY / PN / N / NI |
| **2.6 Was an appropriate analysis used to estimate the effect of assignment to intervention?** |  | Y / PY / PN / N / NI |
| **2.7 If N/PN/NI to 2.6: Was there potential for a substantial impact (on the result) of the failure to analyse participants in the group to which they were randomized?** |  | NA / Y / PY / PN / N / NI |
| **Risk-of-bias judgement** |  | Low / High / Some concerns |
| Optional: What is the predicted direction of bias due to deviations from intended interventions? |  | NA / Favours experimental / Favours comparator / Towards null /Away from null / Unpredictable |

Domain 2: Risk of bias due to deviations from the intended interventions (*effect of adhering to intervention*)

| **Signalling questions** | **Comments** | **Response options** |
| --- | --- | --- |
| **2.1. Were participants aware of their assigned intervention during the trial?** |  | Y / PY / PN / N / NI |
| **2.2. Were carers and people delivering the interventions aware of participants' assigned intervention during the trial?** |  | Y / PY / PN / N / NI |
| **2.3. [If applicable:] If Y/PY/NI to 2.1 or 2.2: Were important non-protocol interventions balanced across intervention groups?** |  | NA / Y / PY / PN / N / NI |
| **2.4. [If applicable:] Were there failures in implementing the intervention that could have affected the outcome?** |  | NA / Y / PY / PN / N / NI |
| **2.5. [If applicable:] Was there non-adherence to the assigned intervention regimen that could have affected participants’ outcomes?** |  | NA / Y / PY / PN / N / NI |
| **2.6. If N/PN/NI to 2.3, or Y/PY/NI to 2.4 or 2.5: Was an appropriate analysis used to estimate the effect of adhering to the intervention?** |  | NA / Y / PY / PN / N / NI |
| **Risk-of-bias judgement** |  | Low / High / Some concerns |
| Optional: What is the predicted direction of bias due to deviations from intended interventions? |  | NA / Favours experimental / Favours comparator / Towards null /Away from null / Unpredictable |

Domain 3: Missing outcome data

| **Signalling questions** | **Comments** | **Response options** |
| --- | --- | --- |
| **3.1 Were data for this outcome available for all, or nearly all, participants randomized?** |  | Y / PY / PN / N / NI |
| **3.2 If N/PN/NI to 3.1: Is there evidence that the result was not biased by missing outcome data?** |  | NA / Y / PY / PN / N |
| **3.3 If N/PN to 3.2: Could missingness in the outcome depend on its true value?** |  | NA / Y / PY / PN / N / NI |
| **3.4 If Y/PY/NI to 3.3: Is it likely that missingness in the outcome depended on its true value?** |  | NA / Y / PY / PN / N / NI |
| **Risk-of-bias judgement** |  | Low / High / Some concerns |
| Optional: What is the predicted direction of bias due to missing outcome data? |  | NA / Favours experimental / Favours comparator / Towards null /Away from null / Unpredictable |

Domain 4: Risk of bias in measurement of the outcome

| **Signalling questions** | **Comments** | **Response options** |
| --- | --- | --- |
| **4.1 Was the method of measuring the outcome inappropriate?** |  | Y / PY / PN / N / NI |
| **4.2 Could measurement or ascertainment of the outcome have differed between intervention groups?** |  | Y / PY / PN / N / NI |
| **4.3 If N/PN/NI to 4.1 and 4.2: Were outcome assessors aware of the intervention received by study participants?** |  | NA / Y / PY / PN / N / NI |
| **4.4 If Y/PY/NI to 4.3: Could assessment of the outcome have been influenced by knowledge of intervention received?** |  | NA / Y / PY / PN / N / NI |
| **4.5 If Y/PY/NI to 4.4: Is it likely that assessment of the outcome was influenced by knowledge of intervention received?** |  | NA / Y / PY / PN / N / NI |
| **Risk-of-bias judgement** |  | Low / High / Some concerns |
| Optional: What is the predicted direction of bias in measurement of the outcome? |  | NA / Favours experimental / Favours comparator / Towards null /Away from null / Unpredictable |

Domain 5: Risk of bias in selection of the reported result

| **Signalling questions** | **Comments** | **Response options** |
| --- | --- | --- |
| **5.1 Were the data that produced this result analysed in accordance with a pre-specified analysis plan that was finalized before unblinded outcome data were available for analysis?** |  | Y / PY / PN / N / NI |
| **Is the numerical result being assessed likely to have been selected, on the basis of the results, from...** |  |  |
| **5.2. ... multiple eligible outcome measurements (e.g. scales, definitions, time points) within the outcome domain?** |  | Y / PY / PN / N / NI |
| **5.3 ... multiple eligible analyses of the data?** |  | Y / PY / PN / N / NI |
| **Risk-of-bias judgement** |  | Low / High / Some concerns |
| Optional: What is the predicted direction of bias due to selection of the reported result? |  | NA / Favours experimental / Favours comparator / Towards null /Away from null / Unpredictable |

Overall risk of bias

| **Risk-of-bias judgement** |  | Low / High / Some concerns |
| --- | --- | --- |
| Optional: What is the overall predicted direction of bias for this outcome? |  | NA / Favours experimental / Favours comparator / Towards null /Away from null / Unpredictable |


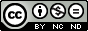


This work is licensed under a [Creative Commons Attribution-NonCommercial-NoDerivatives 4.0 International License](http://creativecommons.org/licenses/by-nc-nd/4.0/).
