# Supplementary material for: Posterior lumbar fusion with and without interbody fusion in isthmic spondylolisthesis: a systematic review and meta-analysis
Source: Neurosurg Rev. 2025 Jul 28;48(1):581. doi: 10.1007/s10143-025-03703-x (PMC12301273; doi:10.1007/s10143-025-03703-x)

Supplementary information 2

**Supplementary figure 1. Forest plot of ODI scores, with RCTs and non-RCTs as subgroups.**


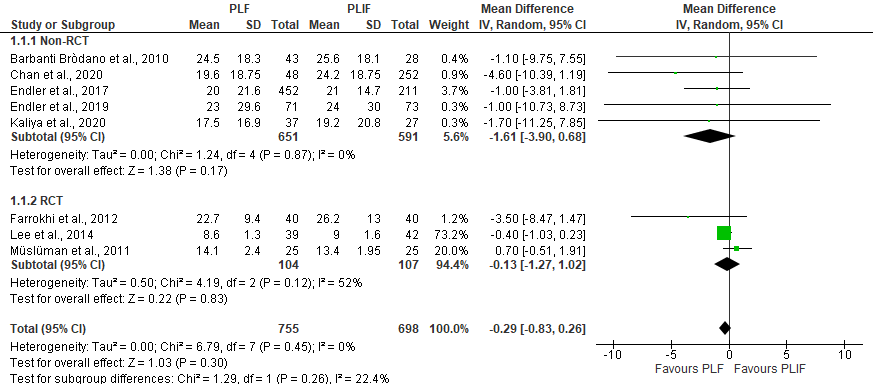


**Supplementary figure 2. Forest plot of complications, with RCTs and non-RCTs as subgroups.**


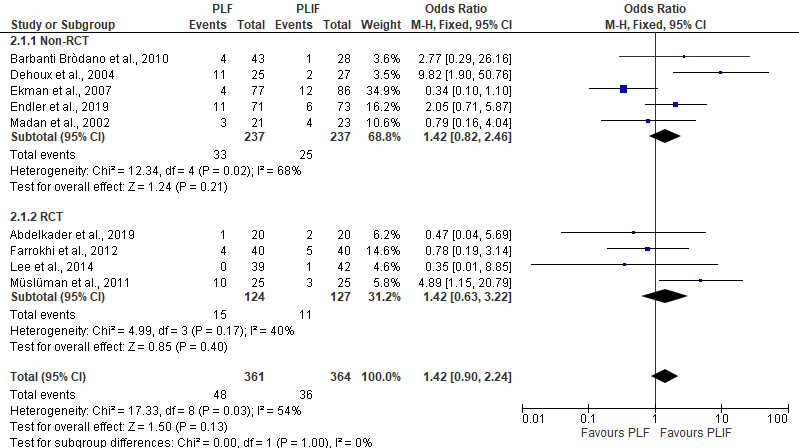

Supplement: Supplementary file 2 — Supplementary Material 2 [file 10143_2025_3703_MOESM2_ESM.docx]
